# Mitochondrial uncoupler DNP induces coexistence of dual-state hyper-energy metabolism leading to tumor growth advantage in human glioma xenografts

**DOI:** 10.3389/fonc.2022.1063531

**Published:** 2022-12-16

**Authors:** Yogesh Rai, Saurabh Singh, Sanjay Pandey, Dhananjay Sah, Raj Kumar Sah, B. G. Roy, Bilikere S. Dwarakanath, Anant Narayan Bhatt

**Affiliations:** ^1^ Division of Molecular and Radiation Biosciences, Institute of Nuclear Medicine and Allied Sciences, Delhi, India; ^2^ Department of Radiation Oncology, Albert Einstein College of Medicine, Bronx, NY, United States; ^3^ Indian Academy Degree College, Bengaluru, India

**Keywords:** Warburg effect, oxidative phosphorylation, glioma tumor, cancer cell metabolism, tumor bioenergetics

## Abstract

**Introduction:**

Cancer bioenergetics is an essential hallmark of neoplastic transformation. Warburg postulated that mitochondrial OXPHOS is impaired in cancer cells, leading to aerobic glycolysis as the primary metabolic pathway. However, mitochondrial function is altered but not entirely compromised in most malignancies, and that mitochondrial uncoupling is known to increase the carcinogenic potential and modifies treatment response by altering metabolic reprogramming. Our earlier study showed that transient DNP exposure increases glycolysis in human glioma cells (BMG-1). The current study investigated the persistent effect of DNP on the energy metabolism of BMG-1 cells and its influence on tumor progression in glioma xenografts.

**Methods:**

BMG-1 cells were treated with 2,4-dinitrophenol (DNP) *in-vitro*, to establish the OXPHOS-modified (OPM-BMG) cells. Further cellular metabolic characterization was carried out in both *in-vitro* cellular model and *in-vivo* tumor xenografts to dissect the role of metabolic adaptation in these cells and compared them with their parental phenotype.

**Results and Discussion:**

Chronic exposure to DNP in BMG-1 cells resulted in dual-state hyper-energy metabolism with elevated glycolysis^++^ and OXPHOS^++^ compared to parental BMG-1 cells with low glycolysis^+^ and OXPHOS^+^. Tumor xenograft of OPM-BMG cells showed relatively increased tumor-forming potential and accelerated tumor growth in nude mice. Moreover, compared to BMG-1, OPM-BMG tumor-derived cells also showed enhanced migration and invasion potential. Although mitochondrial uncouplers are proposed as a valuable anti-cancer strategy; however, our findings reveal that prolonged exposure to uncouplers provides tumor growth advantage over the existing glioma phenotype that may lead to poor clinical outcomes.

## Introduction

A century ago, Otto Warburg and colleagues observed that even in the presence of ample oxygen, tumors have an elevated glucose uptake and lactate production, a phenomenon termed the Warburg effect (1). Warburg suggested that cancer cells increase glycolysis to compensate for the ATP loss due to defective OXPHOS in mitochondria, which converts differentiated cells into undifferentiated forms resulting in unregulated enhanced proliferation as cancer cells (2, 3). Further, oncogenic transformation associated with enhanced ATP synthesis is attained by increased glycolysis rate, providing a metabolic advantage to proliferating cells (4). In addition to catering to the energy needs, an increased influx of the glycolytic intermediates to the pentose phosphate pathway (PPP) generates NADPH and ribose-5-phosphate (precursors for the macromolecular biosynthesis) during the rapid proliferation of the cancer cells (5, 6). While enhanced aerobic glycolysis has long been considered a prominent metabolic signature of cancer, several studies relate tumor cell proliferation and enhanced tumor invasiveness to decreased mitochondrial metabolism and respiratory rate (7). Recent advances in our understanding of cancer cell metabolism have challenged the notion that malignant cells predominantly fulfill their bioenergetic and anabolic needs *via* aerobic glycolysis (8). Accumulating evidence supports the conception that mitochondrial metabolism and OXPHOS influence most oncogenic stages, including malignant transformation and tumor progression (9). While blocking OXPHOS impairs NAD^+^ regeneration preventing tumorigenesis (10), increased ATP production, mitochondrial biogenesis, and OXPHOS have been shown to promote tumor progression and invasion and metastasis of cancer cells (11–13).

Glycolytic suppression has been found to reprogram the energy metabolism in cancer cells towards enhanced mitochondrial OXPHOS (14). This flexible and adapting nature of metabolism confers survival advantages to cancer cells and severely limits the efficacy of approaches targeting glycolysis as a therapeutic strategy in certain cancers (14). The metabolic shift in cancer cells has been recently suggested to depend on nutrient availability with a high degree of metabolic plasticity in progressive malignancies in more than one tumor model (15, 16). Metabolic plasticity allows cancer cells to switch between glycolysis and OXPHOS, suggesting an interplay between these two metabolic states for adaptation in a hostile environment (16–18). Interestingly, some cancers, viz. highly malignant glioma or glioblastoma multiforme (GBM), display a metabolic pattern where elevated glycolysis**^++^** and OXPHOS^++^ coexist, which is associated with the aggressive phenotype and a poor prognosis (19). Based on the above facts, it is tempting to speculate that metabolic plasticity provides survival advantages and confers therapeutic resistance in cancer cells (20). Therefore targeting energy metabolism (glycolysis or OXPHOS) is a primary interest of most cancer metabolism research towards the implication of cancer therapy. In the recent past, the promising outcomes of mitochondrial uncouplers in treating numerous metabolic diseases provided a crucial foundation for exploring their potential in cancer therapy. Multiple studies reported considerable evidence for using different mitochondrial uncouplers as anti-cancer agents (21). Mitochondrial uncouplers cause a short circuit in the electrochemical proton gradient (combined proton gradient and membrane potential) that provides an alternate route to re-entry protons in the mitochondrial matrix and bypass the ATP synthase, eventually resulting in the abrogation of ATP synthesis (21, 22). Multiple pre-clinical research investigations using various uncouplers have shown promising results either *in-vitro* or *in-vivo* in different cancer like neuroblastoma, glioblastoma, leukemia, head, and neck squamous cell carcinoma, adenocarcinoma, breast, ovarian, lung, and colorectal cancer (21). However, the effects of chronic exposure to mitochondrial uncouplers on cancer energy metabolism and tumor growth have not been well studied and remain elusive. The present study aimed to investigate the chronic response of mitochondrial uncoupler, i.e., DNP, which has been shown to induce glycolysis following transient exposure (23) and developed OXPHOS-modified glioma (OPM-BMG) cells from a human glioma cell line (BMG-1) (24). Further, we carried out cellular metabolic characterization in both *in-vitro* and *in-vivo* tumor xenografts to dissect the role of metabolic adaptation in these cells and compared them with their parental phenotype, i.e., BMG-1 cells. Our results showed that OPM-BMG cells have relatively high OXPHOS (OXPHOS^++^) along with high glycolysis (glycolysis**^++^**), which is associated with increased tumorigenic potential (growth and proliferation efficiency). Additionally, cells derived from these tumors also showed increased migration and invasion potential, compared with the relatively low (glycolysis**^+^**) and low (OXPHOS^+^) of its parental phenotype (BMG-1).

## Results

### OXPHOS modification leads to glycolytic up-regulation

**Figure 1:**
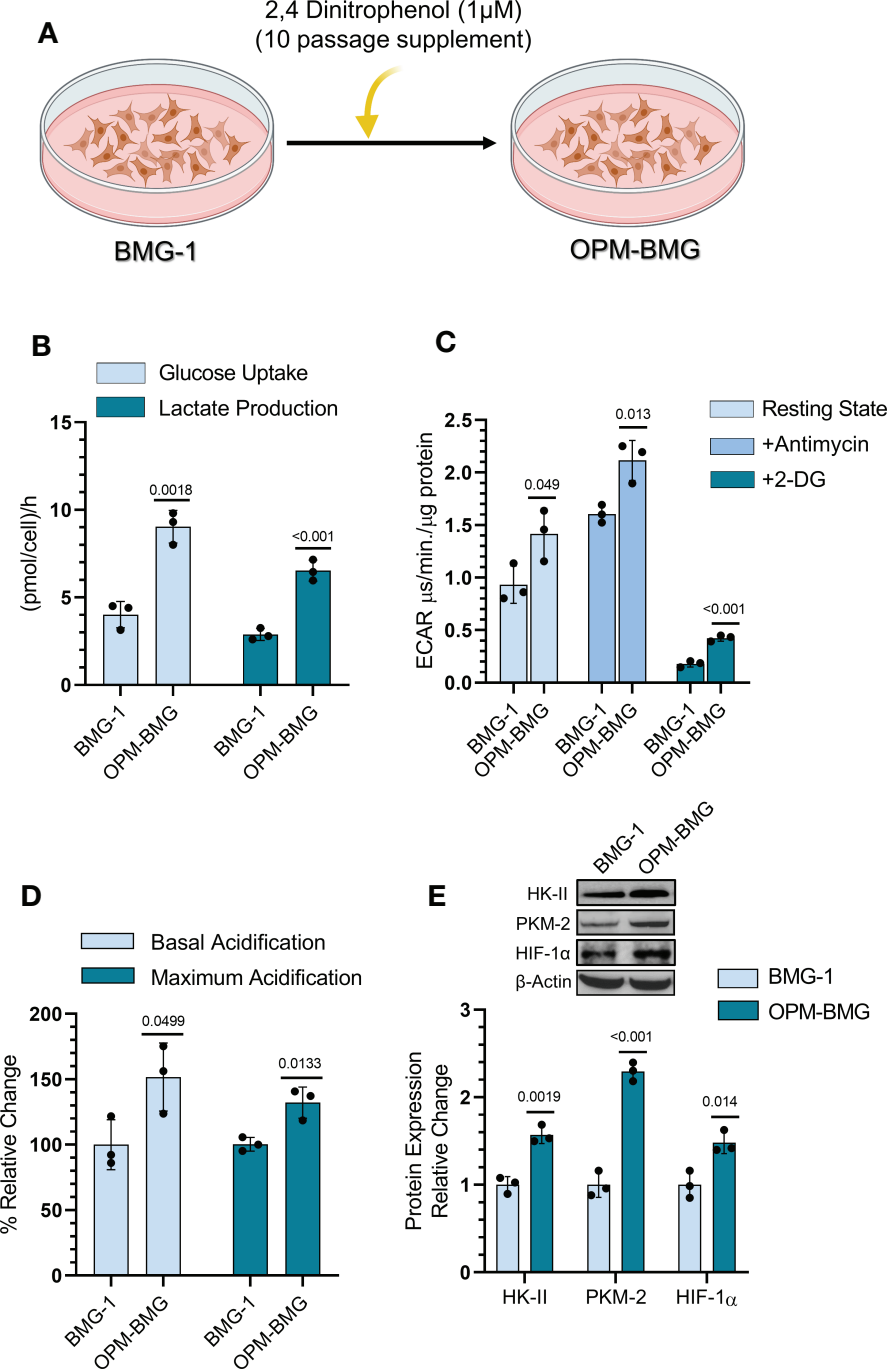
DNP-mediated OXPHOS modification showed glycolytic induction in glioma cells. **(A)** Graphical depiction indicating the methodology opted for establishing the OPM-BMG cell line. **(B)** Glucose consumption and lactate production were estimated from monolayer cells at 4-time intervals of 1 hour (h) each in BMG-1 and OPM-BMG cells (n=3). Data are presented as an average value per hour. **(C)** ECAR was measured as the value of pH Xtra fluorescence lifetime signal (μs/min./μg protein) with or without Antimycin (5 μM) in the indicated cell lines (n=3). **(D)** Information derived from graph C and the overall change in acidification rate of OPM-BMG cells are presented as % relative change with respect to BMG-1. **(E)** Western blot of indicated glycolytic proteins presented with relative quantification among the mentioned cell lines. All data are presented as the mean ± SD; an unpaired two-tailed Student’s t-test was performed to calculate the statistical significance, and *P*-values (BMG-1 versus OPM-BMG cells) are mentioned in the graphs.

BMG-1 cells were treated with DNP (as an ETC modulator), and OXPHOS-modified cells (OPM-BMG) were established *in-vitro* to examine the metabolic plasticity in glioma cells (**Figure 1A**). Further, the cellular characteristics of OPM-BMG cells were compared with their parental phenotype, i.e., BMG-1. Glucose consumption was more than two folds higher in OPM-BMG cells (9 ± 0.75 pmol/cell/h) as compared to the parental BMG-1 cells (4 ± 0.6 pmol/cell/h) (**Figure 1B**). The lactate production was also two-fold higher in OPM-BMG cells showing a substantial increase (6.5 ± 0.48pmol/cell/h) as compared to BMG-1 cells (2.9 ± 0.27 pmol/cell/h) (**Figure 1B**). These observations correlated well with the increased glycolytic flux estimated from the ECAR, which was nearly 50% higher in OPM-BMG (1.41 ± 0.19 μs/min./μg protein) in the resting state compared to BMG-1 cells (0.93 ± 0.14 μs/min./μg protein), (**Figure 1C**). The maximum acidification achieved upon OXPHOS inhibition using Antimycin-A was also increased in OPM-BMG cells (2.11 ± 0.15 μs/min./μg protein) as compared to BMG-1 cells (1.60 ± 0.06 μs·min^−1^·μg^−1^ protein), (**Figure 1C**). Further, an overall relative ECAR revealed an increase in the glycolytic flux of OPM-BMG cells by 50% in the resting state and 32% in case of maximum acidification (+Antimycin), as compared to BMG-1 cells (**Figure 1D**). The critical regulators of the glycolytic pathway, HK-II, PKM2, and HIF-1α that contribute to the metabolic reprogramming in cancer cells (25–27) also showed a profound increase in the OPM-BMG cells in comparison to BMG-1 cells (**Figure 1E**).

These results suggest that OXPHOS modification increases the bioenergetic demand of BMG-1 cells resulting in a metabolic shift towards high glycolysis to support the rapid cell growth in glioma cells.

### ETC modulation results in increased mitochondrial respiration and metabolic hyper-activation

**Figure 2:**
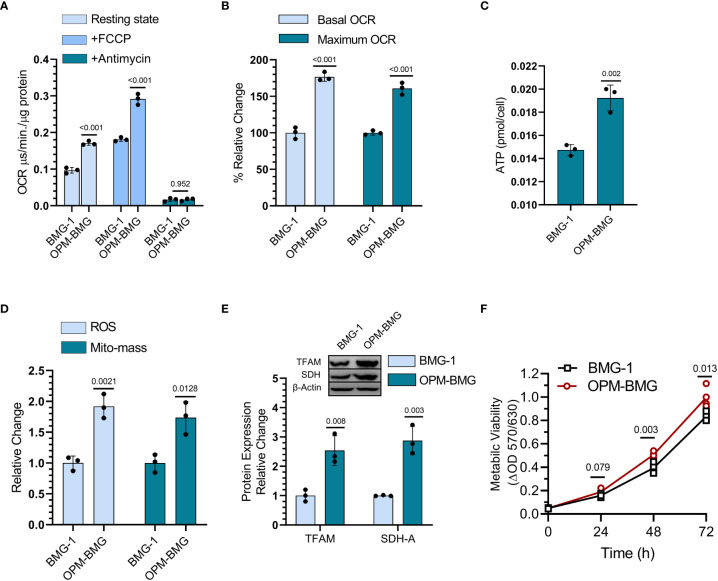
DNP modulates mitochondrial respiration. **(A)** OCR was quantified as the value of MitoXpressXtra fluorescence lifetime signal (μs·/min./μg protein) with or without FCCP (3 μM) in BMG-1 and OPM-BMG cells (n=3). **(B)** The overall change in the OCR showed by OPM-BMG cells is presented as % a relative change with respect to BMG-1 (derived information from graph A). **(C)** ATP content of both BMG-1 and OPM-BMG cells were determined at 24 h post-seeding by ATP bioluminescence assay method, and obtained values were compared among the groups and presented as pmol/cell (n=3). **(D)** Mitochondrial ROS and mitochondrial mass (Mito-mass) were determined using MitoSox Red and MitoTracker Green, respectively. Column graph showing an increase in mean fluorescence intensity (MFI) as a relative change for both fluorescent probes in OPM-BMG cells with respect to BMG-1 cells. **(E)** Immunoblotting of indicated mitochondrial proteins depicting the difference in expression level and densitometric evaluation plotted as relative change among both the cell lines. **(F)** Metabolic viability was evaluated as a mitochondrial function of cell lines by MTT assay (n=4) and presented as a change in MTT value (ΔOD, y-axis) with respect to time (x-axis). Data are presented as the mean ± SD; *P* values (BMG-1 versus OPM-BMG cells) are mentioned in the graphs.

DNP was used to establish an OPM-BMG cell line through ETC modulation, so next, we examined mitochondrial bioenergetics in these cells. The basal level of OCR was nearly two-fold higher in OPM-BMG cells (0.17 ± 0.004 μs/min./μg protein) than in BMG-1 cells (0.096 ± 0.006 μs/min./μg protein), while the maximum OCR (+FCCP) were 0.29 ± 0.012 and 0.18 ± 0.004 μs/min./μg protein respectively (**Figure 2A**). The relative OCR showed a 77% increase in the OPM-BMG cells in the resting state and 61% in the uncoupled (+FCCP) state (**Figure 2B**). The ATP level of OPM-BMG cells was higher and quantified as 0.019 ± 0.0009 pmol/cell compared to BMG-1 (0.014 ± 0.0003 pmol/cell), suggesting an up-regulation of OXPHOS compared with its parental phenotype (**Figure 2C**). An increase in ETC activity was further supported by the enhanced generation of ROS and mitochondrial mass by nearly 85% and 67% in OPM-BMG cells as compared to BMG-1 cells (**Figure 2D**), contributed to the increased aerobic cellular respiration (OXPHOS). The level of mitochondrial biogenesis transcription factor TFAM was analyzed and showed a 2.5-fold increase in OPM-BMG cells compared to BMG-1 (**Figure 2E**), lending further support to the notion that increased OXPHOS is linked to enhanced mitochondrial mass in OPM-BMG cells. We reported earlier that increased mitochondrial biogenesis and succinate dehydrogenase (SDH-complex-II) activity contribute to increased cellular metabolic viability (28). MTT assay showed a significant increase in the metabolic viability of OPM-BMG cells as compared to BMG-1 (**Figure 2F**), which corroborated with a 2.87-fold increase in the level of SDH in OPM-BMG cells (**Figures 2E, F**).

Collectively, these results suggest that DNP-mediated ETC modulation efficiently increases mitochondrial activity, including OCR, and ATP production, with a profound increase in the mitochondrial enzyme (SDH) and biogenesis contributing to enhanced cellular metabolic viability.

### Metabolic hyper-activation results in enhanced cell growth in OPM-BMG cells

**Figure 3:**
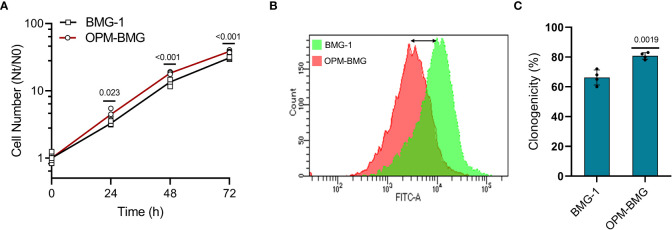
OPM-BMG cells showed an increase in cell growth and clonogenicity. **(A)** Growth kinetics was performed in BMG-1 and OPM-BMG cells (n=4) at indicated time points (x-axis), the exponential increase in the cell number is expressed as Nt/N0 (y-axis). **(B)** The 48-hour CFSE overlay graph shows the right-to-left differential fluorescence shift in cells as a function of cell proliferation. **(C)** Difference in the clonogenicity is presented as percent platting efficiency (%PE; n=4). Data are expressed as the mean ± SD, and *P*-values (BMG-1 versus OPM-BMG cells) are mentioned in the graphs.

Since increased glycolysis is reported as an adaptive mechanism in cancer cells to support the biosynthetic requirements of uncontrolled cell proliferation and improved mitochondrial function results in reduced cell death in cancer cells (29), we studied the kinetics of cell growth and clonogenic efficiency. A shorter population doubling time and a 25% higher cell number at 72 h of growth in OPM-BMG cells (~11 h) compared to BMG-1 cells (15 h) suggested a higher rate and extent of cell growth in the OXPHOS-modified cells (**Figure 3A**). There was also a notable decrease in CFSE fluorescence (left shift in overlay graph), indicating higher cell proliferation in OPM-BMG cells (**Figure 3B**). The clonogenicity of OPM-BMG cells was also increased by 15% than BMG-1 cells (**Figure 3C**).

These results suggest that OXPHOS modification supports enhanced cell growth and clonogenic potential.

### Coexistence of glycolysis^++^ and OXPHOS^++^ increases the *in-vivo* rate of grafted tumor growth

**Figure 4:**
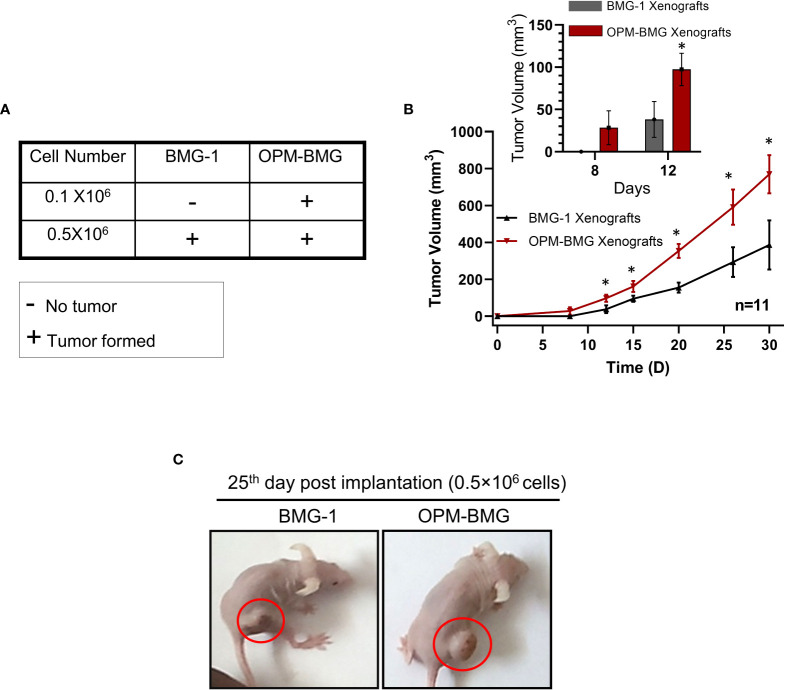
OPM-BMG xenografts showed an increased tumorigenic potential and rapid growth rate. **(A)**. Tumor take analysis was carried out with indicated cell numbers in BMG-1 and OPM-BMG cells (n=5/group). **(B)**. The tumor volume of BMG-1 and OPM-BMG grafted tumors (n=11 mice/group) were plotted, showing an exponential increase in tumor volume (y-axis mm^3^) with the indicated number of days (x-axis) obtained from two independent observations. An inset bar plot compares the first tumor volumes measured in BMG-1 and OPM-BMG xenografts following the development of the initial palpable tumor nodules. **(C)**. Athymic, nude mice images are presented from BMG-1 and OPM-BMG groups, depicting the difference in tumor size captured at the 25^th^-day post-implantation. Data are presented as the mean ± SD; an unpaired two-tailed Student’s t-test was performed to calculate the statistical significance between BMG-1 and OPM-BMG tumor xenografts and presented as *P≤ 0.05 with respect to BMG-1 tumors.

An *in-vitro* study revealed a higher glycolytic flux and OXPHOS in ETC-modulated OPM-BMG cells, which prompted us to investigate the impact of this metabolic adaptation on the formation and progression of tumors xenografted in athymic nude mice. We first performed tumor-take analysis where the mice were subcutaneously inoculated with 0.1×10^6^ and 0.5×10^6^ BMG-1 or OPM-BMG cells (**Figure 4A**). Tumors were formed with the implantation of 0.1×10^6^ and 0.5×10^6^ OPM-BMG cells, whereas tumors were developed with only 0.5×10^6^ BMG-1 cells (**Figure 4A**), suggesting a significant increase in the tumorigenic potential of OPM-BMG cells. The latency period for the initial appearance of palpable tumor nodules was also shorter (4 days) in OPM-BMG compared to ~ 7 days in BMG-1 xenografts. The tumor volume of xenografts was monitored upto 30 days, and considerable differences in the growth were observed from day 15 onwards (**Figure 4B**). In OPM-BMG, tumor volume was quantifiable on the 8^th^ day (4 days post palpability; **Figure 4B** inset) with an average tumor volume of 28 mm^3^ (n=11) and reached 769 mm^3^ by day 30 (**Figure 4B**). On the other hand, in BMG-1 xenografts tumor became measurable on day 12 (5^th^ day after it was palpable; **Figure 4B** inset) with an average of 38 mm^3^ (n=11), reaching a volume of 386 mm^3^ on day 30 (**Figure 4B**), which was nearly two folds lower than the OPM-BMG tumors (**Figure 4B**). Representative images depicting the gross tumor size differences between OPM-BMG and BMG-1 xenografts on day 25 are shown in **Figure 4C**.

These data suggest that the coexistence of glycolysis^++^ and OXPHOS^++^ in OPM-BMG cells contribute to faster tumor growth and reduced latency period.

### OPM-BMG tumor xenografts show glycolysis^++^ and OXPHOS^++^ as metabolic adaptation

**Figure 5:**
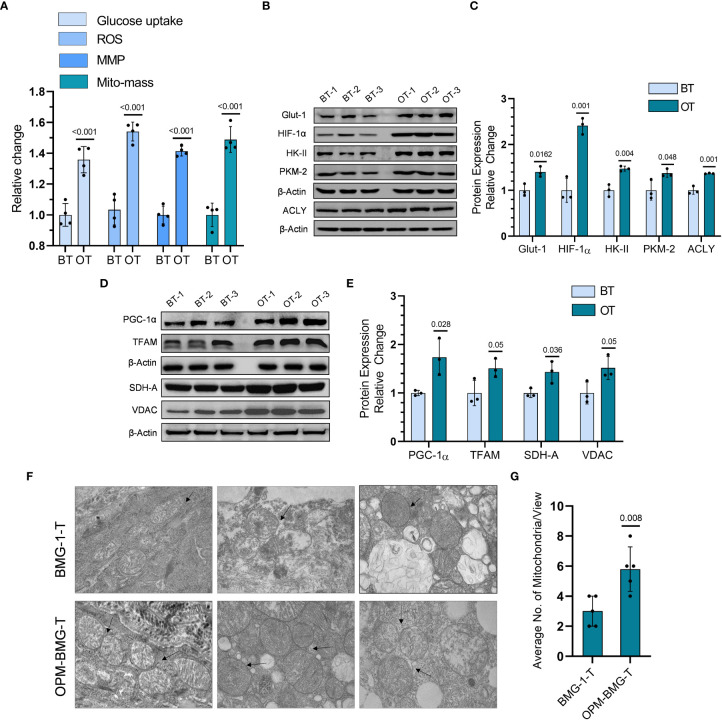
Tumor bioenergetic profile revealed coexisting hyper-metabolism in the OPM-BMG xenografted tumor. **(A)** Glucose consumption, ROS, MMP, and mitochondrial mass (Mito-mass) were estimated in BMG-1 tumor (BT) and OPM-BMG tumor (OT) samples at 25^th^-day post-implantation (n=4mice/group) using fluorescent probes 2NBDG [100μM ], CM-H2DCFDA [10μM], DiOC6 [100nM] and Mito Tracker Green [100nM] respectively and presented as relative change among the BT and OT samples. **(B, C)**. BT and OT samples were subjected to immunoblotting on (the 25^th^ day; n=3 mice/group), and the expression profile **(B)** with the relative densitometric evaluation **(C)** presented among the groups. **(D, E)**. In a similar experimental condition, representative expression of indicated mitochondrial proteins **(D)** and relative change **(E)** are presented in the BT and OT sample (n=3 mice/group). **(F, G)**. TEM images were obtained from different BT and OT samples on the 25^th^ day post-implantation, with arrows indicating the difference in the number of mitochondria/view **(F)** and a bar plot showing the average numbers of mitochondria (n=5; **(G)**. Data are expressed as the mean ± SD; *P*-values (BMG-1 versus OPM-BMG tumor samples) are mentioned in the graphs.

To investigate if the reprogrammed energy metabolism of OPM-BMG cells was also retained in the tumor xenografts, we analyzed the metabolic phenotype, including the levels of certain glycolytic and mitochondrial regulators in the tumor tissues. OPM-BMG tumors showed a significant increase in glucose uptake, ROS level, MMP, and mitochondrial mass compared to their parental phenotype (**Figure 5A**). Additionally, in line with the *in-vitro* observations, OPM-BMG xenografts also showed a profound increase in critical glycolytic protein expression levels, including HK-II, GLUT-1, PKM-2, and HIF-1α, as compared to BMG-1 tumors (**Figures 5B, C**). Interestingly, we also observed overexpression of ATP-citrate lyase (ACLY) in OPM-BMG tumor xenografts (**Figure 5C**), an established positive regulator of glycolysis in glioblastoma (30) suggestive of increased fatty acid biosynthesis. Additionally, in OPM-BMG tumors, the upregulated level of mitochondrial biogenesis regulatory proteins PGC-1α (~1.73 fold) and TFAM (~1.5 fold), compared to BMG-1 (**Figures 5D, E**), confirmed an increase in mitochondrial mass and the induction of mitochondrial biogenesis in OPM-BMG tumor tissue. Moreover, augmented mitochondrial mass in the OPM-BMG tumor tissues was visually supported by the images obtained from transmission electron microscopy (TEM) and showed a considerable increase in the number of mitochondria compared to BMG-1 tumors (**Figures 5F, G**). Further, the level of succinate dehydrogenase-A (SDH-A), the catalytic subunit of succinate-ubiquinone oxidoreductase (complex-II), and voltage-dependent anion channel (VDAC) showed a substantial increase in OPM-BMG tumors (**Figures 5D, E**). An up-regulation of SDH-A and VDAC observed along with the enhanced mitochondrial ROS, and MMP (by ~1.5 fold and ~1.42 fold, respectively; **Figure 5A**) implied an upregulated functioning of ETC in OPM-BMG tumors compared to its parental phenotype.

These results suggested that increased co-expression of glycolytic and mitochondrial regulators contributes to the aggressive tumor phenotype in OPM-BMG cells.

### OPM-BMG tumor xenografts showed increased neovascularization, migration, and invasion potential

**Figure 6:**
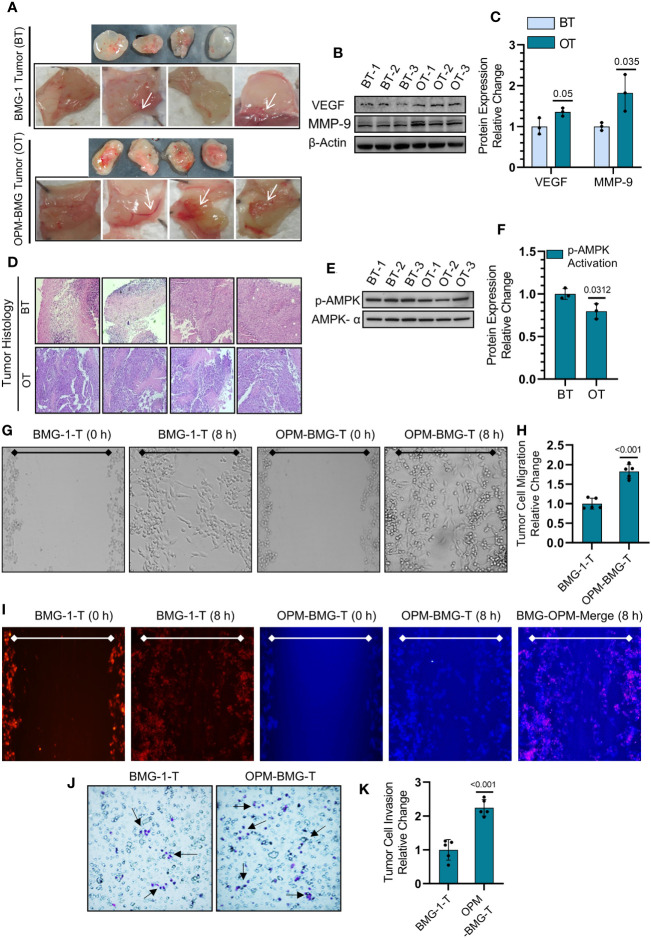
OPM-BMG tumors and derived cells showed aggressive tumor characteristics. **(A)** Photomicrographs of Matrigel plug depicting the angiogenesis in BMG-1 and OPM-BMG xenografted tumor (cell density 0.5×10^6^) at 7^th^-day post-implantation (n=4/group). **(B, C)**. Change in the expression level of proangiogenic proteins VEGF and MMP-9 **(B)** and presented as the relative change in the protein expression among BMG-1 tumor (BT) and OPM-BMG tumor (OT) samples (n=3 mice/group; **(C)**. **(D)** Histological examination of intra-tumoral compartments after H&E staining presented on the 25^th^ day of post-tumor implantation (n=4 mice/group). **(E, F)**. Metabolic energy stress marker proteins AMPK-α and its activated form p-AMPK-α were examined in BT and OT samples (n=3 mice/group; **(E)** and presented as the relative change in the indicated tumor samples **(F)**. **(G, H)**. Migration of tumor-derived cells, i.e., BMG-1-T & OPM-BMG-T, were monitored (after 8 h) in response to the mechanical scratch wound in unstained cells (n=5), followed by quantitative evaluation presented as relative change among the groups **(H)**. **(I)** Migration potential in BMG-1-T and OPM-BMG-T cells were examined in co-culture of separately stained cells using CTR (BMG-1-T cells) and CTB (OPM-BMG-T cells) fluorescent probes, respectively (n=5). Differential images of cell movement were captured in the respective emission channel of probes, while merge staining is presented for better appreciation. J&K. Transwell invasion assay was performed by analyzing the differential chemotactic movement of BMG-1-T and OPM-BMG-T cells. In photomicrographs, arrows indicate the different stained cell populations in both groups **(J)**; cells were further counted from five random field views and presented as relative change among the groups **(K)**. The bar plots are the mean ± SD; *P*-values (BMG-1 versus OPM-BMG tumor samples) are mentioned in the graphs.

We next employed an *in-vivo* Matrigel plug assay to determine the effect of this metabolic modulation on the process of tumor-associated neo-angiogenesis that plays an essential role in facilitating tumor growth and progression. On the 7^th^ day post-implantation, OPM-BMG plugs showed a prominent developing vessel-like network (neo-angiogenesis), indicating increased tumor vascularity at the edges and surrounding the tumor, compared to a relatively lesser neo-vasculature in BMG-1 Matrigel plugs (**Figure 6A**). We also observed a significant increase in the expression levels of VEGF and MMP-9, confirming increased angiogenesis in OPM-BMG tumor xenografts (**Figures 6B, C**). Moreover, histological examination of intra-tumoral heterogeneity using H&E (hematoxylin & eosin) staining showed a condensed heterochromatin pattern (hematoxylin staining) in OPM-BMG tumors, compared to relatively sparse staining in BMG-1, indicating increased tumor proliferation efficiency in OPM-BMG tumor xenografts (**Figure 6D**). Relatively slower growth of BMG-1 xenografts compared to the OPM-BMG tumors was accompanied by a ~ 20% increase in the levels of p-AMPK (**Figures 6E, F**), an activated form of AMP-activated protein kinase (AMPK), in BMG-1 tumors, indicating that metabolic stress eventually reduces the ATP consuming anabolic processes required for cell growth and proliferation (31). Moreover, in both the xenografts, the metastatic progression was confirmed by *in-vitro* migration potential and invasion in tumor-derived cells (from the tumors grown *in-vivo*), (**Figures 6G, K**). The effect of metabolic modulation on migration was evaluated by the scratch assay. In response to the mechanical scratch wound, OPM-BMG tumor cells showed a significant increase of nearly 1.8-fold cell migration than its parental phenotype BMG-1, observed as early as 8 h post-scratching (**Figures 6G, H**). Additionally, migration potential was also monitored in co-culture of both the tumor cells stained separately using fluorescent probe cell tracker red (for BMG-1) and cell tracker blue (for OPM-BMG). An increase in the cell movement of OPM-BMG cells was validated by enhanced fluorescence signal (Blue>Red) captured in the respective emission channel and merged images (**Figure 6I**) as well. Further, the invasive potential evaluated using the Boyden chamber assay revealed a significantly higher degree (~2.3 fold) of invasiveness of OPM-BMG cells compared to BMG-1 tumor cells (**Figures 6J, K**). These results suggest that modification of oxidative phosphorylation enhances angiogenic potential, i.e., the endothelial phenotype in OPM-BMG tumor xenografts, accompanied by increased tumor proliferation efficiency, supported by high migration and invasion of tumor-derived cells than its parental phenotype.

## Discussion

Mitochondrial uncouplers have received good attention in the recent past for their potential to cure cancer. However, the chronic effects of mitochondrial uncouplers on tumor cell metabolism and its consequences on tumor progression still argue for more exploration. Warburg phenotype has long been recognized as a signature characteristic of tumor metabolism; nevertheless, emerging evidence has highlighted the coexistence of enhanced glycolysis and oxidative phosphorylation in cancer and emphasized the importance of this phenotype on the biological behavior of tumors, including its strong correlation with the aggressive growth of glioblastoma multiforme (19, 32). In the present study, mild mitochondrial uncoupling caused by DNP in the BMG-1 glioma cells resulted in the reprogramming of metabolism wherein an increase in the glycolytic flux coexisted with an increase in OXPHOS as well, designated as OPM-BMG cells (**Figures 1**, **2**). The increase in glycolytic flux appeared to be driven by the up-regulation of HK-II, HIF-1α as reported by us earlier (23) as well as PKM-2, some of the critical determinants of the glycolytic pathway (27). Interestingly, the OPM-BMG cells were also associated with increased mitochondrial biogenesis and metabolic viability as compared to the parental BMG-1 cells (**Figures 2E, F**). We have previously shown that the mitochondrial uncoupling using DNP contributes to increased OCR and ATP production, besides conferring resistance against radiation-induced cell death (33). Understanding from our previous studies (23, 33) and the results of the present study confirmed that mild mitochondrial uncoupling has two consequences: one is increased glycolysis, and the second is an increase in the mitochondrial mass. In the context of the present study, the simultaneous increase of glycolysis and OXPHOS is an adaptive response of established phenotype, i.e., OPM-BMG. It is pertinent to note that DNP treatment leads to an increase in the functional mitochondria that tend to require more fuel substrate for ETC functioning, necessitating increased glycolysis to maintain the cellular bioenergetics. Therefore, tumor cells with reprogrammed metabolism comprising high glycolysis^++^ and OXPHOS^++^ like OPM-BMG cells would not only be efficient in proliferation but could also be resistant to therapies. Retention of this phenotype under *in vivo* conditions, as revealed by the increased rate of tumor progression in OPM-BMG xenografts compared to BMG-1 (**Figure 4B**), suggests the robustness of this phenotype that is not attenuated by factors associated with the tumor microenvironment. Further, the rapid tumor growth in OPM-BMG xenografts was associated with an increase in the expression of pro-angiogenesis factors like VEGF and MMP-9 (**Figures 6A–C**), suggestive of stimulated neovascularization (34, 35) that appears to be driven by the increased ROS generation (**Figure 5A**) (33). Increased mitochondrial activity results in enhanced leakage of mitochondrial ROS, and it is evident that ROS plays an important role in stabilizing HIF-1α and associated signaling (36). A substantial increase in the HIF-1α expression observed in OPM-BMG cells *in-vitro* (**Figure 1E**) and tumor tissues (**Figures 5B, C**) compared to BMG-1 may be responsible for promoting proangiogenic pathway and neovascularization (37). Hypoxia in tumors upregulates glycolysis resulting in increased lactate production which is metabolized aerobically by normoxic cells in the mitochondria, allowing them to evade anti-angiogenesis therapies (38). Increased expression of glycolytic and OXPHOS regulators in the OPM-BMG tumors observed here (**Figures 5B–E**) indicated the retention of the metabolic phenotype of the OPM-BMG cells in the tumor microenvironment of the xenograft. Further, the enhanced migratory and invasive potential of cells obtained from OPM-BMG tumors indicated higher metastatic potential suggestive of an aggressive phenotype. Although the role of glycolysis is well established in metastatic progression mediated by HIF-1α, HK-II, and PKM2 (39–41), enhanced OXPHOS activity has also been shown to contribute to higher metastatic potential (11, 42). A strong association of migration and invasive properties with PGC-1α, a key regulator of mitochondrial biogenesis and OXPHOS, besides ROS, has been recently shown in breast cancer cells (11). A significant up-regulation of HIF-1α, HK-II, and PKM2 in addition to ROS, as well as both the enhanced biogenesis transcription factors PGC-1α and TFAM, were observed in OPM-BMG compared to BMG-1 tumor (**Figure 5**) substantiates the impact of high glycolysis^++^ and OXPHOS^++^ in the higher invasive potential seen in these tumor-derived cells (**Figures 6J, K**). Further, an increase in tumor growth and proliferation efficiency in OPM-BMG tumors (**Figure 4**) correlated well with the downregulation of activated AMPK (p-AMPK; **Figures 6E, F**) (43) coupled with HK-II, Glut-1, and PKM-2 (**Figures 5B, C**), leading to glycolytic up-regulation compared to its parental phenotype. ACLY expression is known to contribute to the upregulated fatty acid biosynthesis and glycolysis (44, 45) and play a role in the radio-resistance of nasopharyngeal carcinoma (46). A higher level of ACLY seen in OPM-BMG tumors compared to the parental BMG-1 tumors suggests that this metabolic phenotype would also lead to therapeutic resistance. Recent system biology studies have proposed that the coexistence of upregulated HIF-1α and activated AMPK are indicative of increased glycolysis and OXPHOS in cancer cells with a hybrid state of metabolism (17). However, the OPM-BMG tumors had a marginally lower level of p-AMPK but showed a substantial increase in the HIF-1α (**Figures 5B, C**, **6E, F**), suggestive of a metabolic adaptation in transition. The reduced level of p-AMPK obtained in OPM-BMG tumor tissues also indicates that it has reduced energetic stress due to enhanced aerobic glycolysis and OXPHOS.

Increased MMP-9 and ROS levels (**Figure 5A**) seen in OPM-BMG tumors without a significant loss of AMPK activity appear to support the functional restoration of mitochondria leading to an increased vasculature that could contribute to higher growth and aggressive behavior. Oxidative stress is suggested as a critical player in the increased vasculature and higher tumor growth rate (36). Up-regulation of mitochondrial membrane protein VDAC and the critical glycolytic regulator HK-II (**Figures 5B, D**) in OPM-BMG tumors point toward the advantage of this metabolic adaptation. The binding of HK to the outer membrane of mitochondria in association with VDAC is required for ATP’s easy accessibility to initiate the glycolytic reaction (47, 48). Interaction of HK-II and VDAC in cancer cells suggests a strong interconnection between glycolysis and mitochondrial respiration (49), indicating metabolic symbiosis as seen here in the OPM-BMG tumor xenografts (**Figure 5**). Higher levels of both HK-II and VDAC seen in OPM-BMG cells and tumor samples (**Figures 5B–E**) also support the existence of high glycolysis**^++^** and OXPHOS**^++^** that could contribute to the aggressive tumor phenotype in OPM-BMG as compared to the BMG-1tumor xenografts. A significant increase in the PGC-1α, TFAM, and SDH levels in the OPM-BMG tumor xenografts compared to BMG-1 confirmed an increase in mitochondrial biogenesis in OPM-BMG (**Figure 5D–G**).

**Figure 7:**
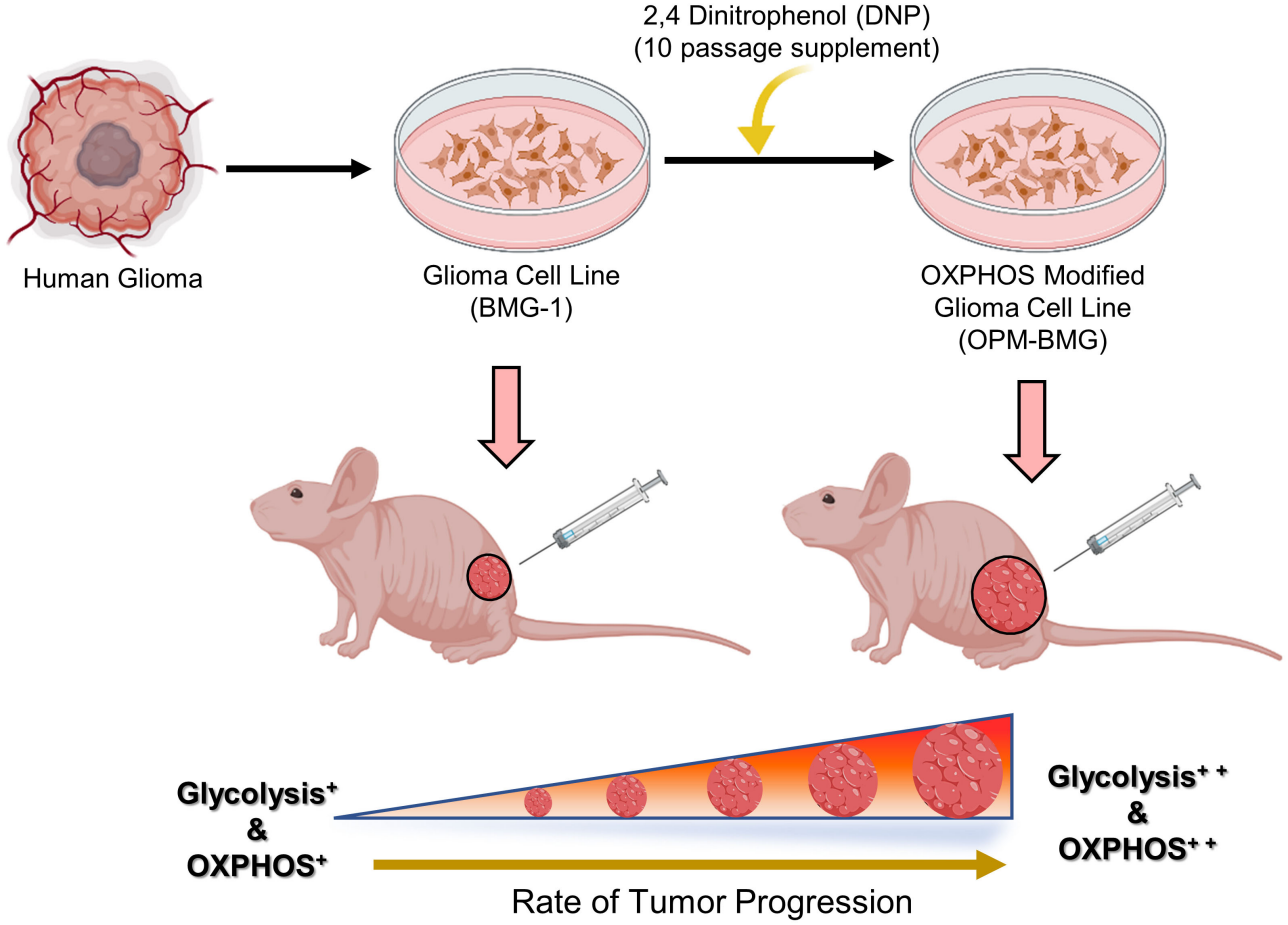
(Summary Figure): Graphical illustration indicates the prolonged effect of mitochondrial uncoupler on the energy metabolism of glioma cells. The human glioma-derived cell line BMG-1 was treated with DNP to modify the electron transport chain, which resulted in a phenotype of dual-state hyper-energy metabolism characterized by increased glycolysis^++^ and OXPHOS^++^ compared to its parental phenotype (BMG-1) of low glycolysis^+^ and OXPHOS^+^. Tumor xenograft investigation shows that OPM-BMG cells with dual-state hyper-energy metabolism have increased tumorigenic potential and accelerated tumor growth, compared to its parental phenotype. (The graphical illustration was generated using Biorender software).

In conclusion, our findings indicate that a chronic response to mild mitochondrial uncoupler is responsible for the coexistence of high glycolysis^++^ and OXPHOS^++^ as part of the reprogramming of energy metabolism that occurs throughout the development of OPM-BMG xenografts. Moreover, the symbiotic relationship between glycolysis and OXPHOS provides growth and survival advantages during the increased rate of tumor progression and also plays a role in acquiring an aggressive glioma tumor phenotype (Summary **Figure 7**). The current work demonstrates that prolonged exposure to mitochondrial uncoupler as an anti-cancer medication has the potential to change the treatment response by promoting an aggressive tumor phenotype. Even though further studies are required to understand the impact of this metabolic phenotype on therapeutic resistance, nonetheless, concerning tumor cell metabolism, this study highlights the chronic exposure limitation of mitochondrial uncouplers that should be taken into consideration. Moreover, the study warrants further investigations on approaches exploiting both glycolytic and OXPHOS inhibition or disruption of the symbiotic relationship between the two metabolic pathways to treat aggressive glioma tumors for better clinical outcomes. In the same vein, additional research is also required in other model systems to bolster our hypothesis of a mitochondrial uncoupler linked to the dual role of energy metabolism in the aggressive tumor phenotype.

## Materials and methods

### Reagents and chemicals

Cell growth medium, Dulbecco’s Minimum Essential Medium (DMEM) low glucose (Cat# D5523-10X1L), Penicillin G, Streptomycin, and Nystatin were all purchased from Sigma Chemicals Co. (St Louis, U.S.A.). CM-H2DCFDA, DiOC6, Mito-Tracker Green FM, Cell Tracker™ Red (CTR), Cell Tracker™ Blue (CTB), and CFSE were obtained from Molecular Probes (Eugene, U.S.A.). Matrigel (HC) was procured from BD Biosciences (Bedford, MA). Primary antibodies HK-II (Cat# SC-6521), PKM-2 (Cat# SC-65176), TFAM (Cat# SC-23588), Glut-1 (Cat# SC-7903), purchased from Santa Cruz Biotechnology (CA); HIF-1α (Cat# 3716S), SDH-A (Cat# 11998S), VEGF-R2 (Cat# 2479 S), MMP-9 (Cat# 13667S), AMPK-α (Cat# 2532S), p-AMPK-α (Cat# 2535 S), ACLY (Cat# 4332S), PGC-1α (Cat# 2178S), VDAC (Cat# 4866S) procured from Cell Signaling Technology (Danvers, MA, USA); and β-Actin (612657) obtained from BD Biosciences (CA), whereas Horseradish Peroxidase (HRP) conjugated secondary antibodies were procured from Thermo Fisher Scientific (USA).

### Sources of tumor cell lines

The human brain malignant glioma (BMG-1; diploid, wild type p53) cell line used in this study (BMG-1; diploid, wild type p53) was previously established by us (24) at the National Institute of Mental Health and Neurosciences, Bangalore, India. Whereas OPM-BMG cell line was established by modulation of the ETC using mitochondrial uncoupler DNP (1µM) as a culture supplement, added for 10 passages in BMG-1 cells. DNP (1µM) was used in routine passaging to maintain this cell line as OPM-BMG cells. Growth medium DMEM low glucose supplemented with 5% FBS, HEPES, and antibiotics, were used to maintain the cell lines as monolayers in 60 mm tissue culture Petri dishes (PD-60; BD Falcon, U.S.A.) at 37°C in a humidified incubator with 5% CO_2_. All cell culture experiments were performed with the exponentially growing cells.

### Ethics statement

Throughout the entirety of the study, mice were provided care in accordance with the guidelines outlined earlier (50). All animal experiment protocols used in the present study were reviewed and received ethical approval by the Institute of Nuclear Medicine and Allied Sciences (Institutional Ethical Committee approval; INM/IAEC/2017/20 and INM/IAEC/2018/21).

### Growth kinetics, MTT, and clonogenic assay

To study growth kinetics, 0.075×10^6^ cells from each group were seeded in PD-35 and counted at different time intervals (post cell seeding) using a Neubauer improved counting chamber (Paul Marienfeld GmbH & Co. KG, Germany) and a compound light microscope (Olympus CH30, Japan). Obtained cell numbers at (Nt; 24, 48, and 72 hours) were normalized with the cell density (at N; 0 hours), and the graph was plotted as Nt/N0. Cells were seeded in 96-well plates at a density of 0.005×10^6^ cells per well for the determination of metabolic viability using MTT assay. At the time points specified in the experiment, MTT working concentration of (0.5mg/ml) was added, and cells were incubated in the dark for 2 hours at 37°C in a CO_2_ incubator. After incubation medium was removed, and DMSO (200 µl/well) was added to dissolve the formazan crystals formed by the cells. Finally, absorbance was measured at 570 nm with a reference wavelength of 630 nm on a Multiwell plate reader (Biotech Instruments, USA). For the clonogenic assay, 100 cells from each group were seeded in PD-60 and incubated for 7-8 days in a CO_2_ incubator to allow the formation of macroscopic colonies. After cell washing in PBS and staining with crystal violet, colonies with at least ≥ 50 cells were counted. Finally, normalizing the number of colonies formed with the number of cells seeded, the plating efficiency (PE) of both cell lines was determined as percent clonogenicity.

### CFSE cell proliferation assay

Cell proliferation assay was performed using CFSE (5-(and-6)-Carboxyfluorescein Diacetate, Succinimidyl Ester) according to manufacturer protocol. Briefly, cells were incubated with 5µM CFSE in a medium supplemented with 2% serum at room temperature for 20 minutes (with continuous rolling), followed by seeding with a density of 0.075×10^6^ in PD-35 and kept at 37°C in a CO_2_ incubator. After 48 hours, cells were terminated CFSE uptake was analyzed using a flow cytometer (BD, FACS Aria Tm III Cell Sorter, USA).

### Estimation of glucose consumption and lactate production

Glucose consumption and lactate production were performed 24 h post-seeding as our previously described method (23). Both BMG-1 and OPM-BMG cells were allowed to grow in HBSS medium for a period of four hours. At the end of each hour, 100 µl of the medium was removed and placed in a freezer until further examination. A glucose-oxidase method-based kit (Tecnicon RA-500 auto-analyzer) and lactate estimation kit (Randox; Cat. No.-LC2389) were used to estimate the unused amount of glucose and lactate production, respectively. The obtained data were normalized by cell number, and the graph was plotted as pM/cell/h. In tumor tissue, glucose consumption was estimated using fluorescent glucose analog 2-NBDG. Tissue samples were minced in PBS and filtered with a cell strainer (70M), then 2-NBDG (100μM ; 20 min. at 37°C) was added to the cell-PBS suspension, followed by PBS washing and acquisition on LSR-II flow cytometer (BD, Mountainview, CA, USA).

### ATP measurement

Both BMG-1 and OPM-BMG cell lines were seeded at a density of 0.3×10^6^ in-35 to estimate cellular ATP content. Cells were harvested 24 hours after seeding, and an ATP bioluminescence assay was performed using the ATP bioluminescence assay kit (Invitrogen USA) as directed. After normalization from the respective group cell number, an estimated cellular ATP content presented as pmol/cell.

### Estimation of ECAR and OCR

The ECAR and OCR were determined using the pH Xtra glycolysis and MitoXpressXtra oxygen consumption assay kits (Agilent, Chicopee, MA, USA), respectively. Cells were seeded into 96-well plates at 0.08 × 10^6^ cells/well in the complete growth medium. OCR was performed as described earlier (33), whereas to perform the ECAR assay, the culture plate was placed in a CO_2_-free incubator at 37°C with 95% humidity for two hours. Wells were gently washed two times with respiration buffer, and 10 µl of pH Xtra reagent was added to each well containing 90 µl of buffer medium. Antimycin A (5 µM) was used to achieve maximum acidification, and 2-DG (50 mM) was applied as a negative control. Fluorescence intensity signals for ECAR and OCR dual read TR-F (time-resolved fluorescence), fluorescence measurements 380 nm/645 nm (ex/em.) were repeatedly recorded every 5 minutes interval over 100 minutes using Synergy H1 Hybrid Multi-Mode Microplate Reader (BioTek Instruments, USA). Dual read TR-F settings were maintained for ECAR with integration window 1 (100 μs delay D1; 30 μs measurement time W1) and integration window 2 (300 μs delay D2; 30 μs measurement time W2). After being normalized with the respective protein content, the measured TR-F intensity signals of ECAR and OCR were converted into lifetime signals (μs) as described earlier (33) and presented as μs·min^−1^ μg^−1^ protein.

### Tumor study

The tumor xenograft study was conducted using six-week-old female athymic nude (BALB/c) mice (n=11/group) and acclimatized for one week before study commencement. For tumor cell inoculation, cells were grown to 70-90% confluency, and the medium was replaced with fresh growth medium 24 hours prior to harvesting by trypsinization. 100μl of inoculum containing 0.1x10^6^ or 0.5×10^6^ cells were injected into each mouse of respective groups (BMG-1 and OPM-BMG). To determine the tumor’s volume, the tumor’s length, width, and height were initially measured with Vernier calipers and finally estimated using the formula 1/2× (Length×Width×Height). On the 25^th^day after implantation, mice were sacrificed, and tumor specimens were dissected, fixed with 10% buffered formalin, and embedded in paraffin for tumor histology. Haematoxylin and Eosin (H&E) stains were applied to paraffin sections, and intra-tumoral proliferative compartments were examined, followed by a comparative analysis between BMG-1 and OPM-BMG groups. The digital images of histology slides were visualized with the bright-field microscope(IX 51, Olympus, Japan) using 10x (objective)×10x (eyepiece) magnification.

### *In-vivo* matrigel plug assay

The difference in the tumor-associated angiogenesis was determined using Matrigel, which mimics the physiological cell matrix. Briefly, 250 µL of unpolymerized Matrigel (~10 mg/mL) was mixed with 250 μL of serum-free medium containing BMG-1 and OPM-BMG cells (0.5×10^6^ cells/mice). Finally, each mouse was subcutaneously injected with a 500 µL volume of tumor cells containing matrigel mix at the right flank of the mouse (n=6/group). On the 7^th^ day post-implantation, animals were sacrificed, and the plugs were dissected and photographed.

### Establishment of tumor-derived cells

Solid tumors were dissected into small pieces using a sterile scalpel, and tissue was minced in PBS, followed by filtration using a cell strainer (pore size 70 µM). The filtered cell suspension was further processed for PBS washing and centrifugation (1500g) steps, repeated thrice. The cell pellet was resuspended in a complete growth medium in a T-25 flask and kept at 37°C in a CO_2_ incubator. Cells were monitored for doubling and confluency; the medium was replaced routinely to remove dead cell floaters. Finally, experiments were performed with exponentially growing cells after 3-5 *in-vitro* passaging.

### *In-vitro* wound-healing assay (Scratch assay)

Cell migration was performed in tumor-derived cells as the previously described method (51). Briefly, cells were seeded in 24 well plates with a cell density of 0.04×10^6^ cells/well and allowed to attain confluency. A mechanical “wound” was created by scraping with a 200-µl sterile pipette tip. As cells traveled from the intact zones into the scratched region, cell migration was monitored under an inverted bright-field microscope (Olympus, Japan) using a 10 x (objective) × 10X (eyepiece) magnification. In a different experiment, cell movement was also examined using fluorescent probes. Both BMG-1 and OPM-BMG cells were stained separately with 5µM each CellTracker™ Red and CellTracker™ Blue, respectively, in a serum-free medium, followed by co-culture in a complete growth medium. Fluorescence images were captured under blue and red emission channels for respective dyes. A minimum of 5 random field views of cell movement were enumerated, and average data was presented as the relative change in the tumor cell migration.

### *In-vitro* invasion assay

The transwell invasion assay was performed to analyze the differential chemotactic responses in tumor-derived cells (BMG-1 and OPM-BMG) as the previously described method (51). The 24 well plates were used with Hanging Millicell inserts (Millipore) of 8-μm polyethylene terephthalate membrane filters. Cells were seeded with a density of 0.05×10^6^ cells/well in the upper chamber with 200 μl of serum-free medium, whereas 500 μl of complete growth medium with 5% FBS was added to the bottom chamber. The plates were incubated at 37°C in a humidified environment with 5% CO_2_ for 24 hours. The non-migratory cells remaining in the upper chamber were removed using a cotton swab, and cells in the lower chamber were fixed in 4% paraformaldehyde, followed by permeabilization with methanol and Giemsa staining. Stained cells on the lower surface of the membrane were counted in five random fields under an inverted microscope using 10 x (objective) × 10x (eyepiece) magnification. Data were obtained as the average number of invading cells from different field views and plotted as differential fold change between BMG-1 and OPM-BMG cells.

### Estimation of ROS, MMP, and mitochondrial mass

To stain tumor tissues, samples were initially minced in PBS and filtered through a cell strainer (Millipore, pore size 70 µM). Further tissue and cell samples were processed in a similar way with the appropriate probes. Both mitochondrial and intracellular ROS was measured using CM-H2DCFDA (10 μM) and MitoSox Red (5μM), respectively. In a similar experimental condition, MMP and mitochondrial mass were estimated using fluorescent probes DiOC6 (100 nM) and Mito-Tracker Green FM (100 nM), respectively. As a final step, cell lines and tissue samples were washed and resuspended in 500 μl of PBS for acquisition in respective FITC and PE channels on an LSR-II flow cytometer (BD, Mountainview, CA, USA).

### Immunoblotting

For immunoblotting whole cell lysates or tumor tissue samples were lysed in RIPA lysis buffer, and western blotting was performed as the previously described method (33). Names and details of primary antibodies, including glycolytic and mitochondrial proteins, are mentioned in the chemicals and reagent section. The protein expression analysis specified in the experiments involved the blot densitometry evaluation and normalization with the corresponding loading control (β-actin). Further data from the BMG-1 and OPM-BMG cell lines/tumor samples were compared and presented as protein expression relative change.

### Transmission electron microscopy

For the TEM study, Trump’s solution (4% formaldehyde in 0.1M phosphate buffer) was used to fix tumor tissue samples overnight at room temperature, followed by secondary fixation in 1% osmium tetroxide solution and 1% aqueous uranyl acetate. Tissues were embedded in epoxy resin after being dehydrated in ethanol concentrations (50, 70, 90, and 100%). Thin sections with a thickness of 0.1 micrometers were collected on copper grids and post-stained with lead citrate and uranyl acetate. Further images were viewed at 120 kV with a JEOL 2100 transmission electron microscope (JEOL USA, Peabody, MA). For each tumor sample, the ten randomly selected mitochondrial profiles were scored based on their appearance: regular elongated (>3μm long), ovoid (0.5μm in diameter), teardrop-shaped, or mitochondria-on-string (MOAS).

### Statistical analysis

Unless otherwise stated, all the experiments were performed at least three times, with each condition analyzed in triplicates. Data are expressed as means ± SD. The statistical significance was calculated by unpaired two-tailed Student’s t-test using GraphPad Prism software (version 8), and values *p* ≤ 0.05 was considered statistically significant.

## Data availability statement

The raw data supporting the conclusions of this article will be made available on request by the authors, without undue reservation.

## Ethics statement

The animal study was reviewed and approved by Institute of Nuclear Medicine and Allied Sciences (Institutional Ethical Committee approval; INM/IAEC/2017/20 and INM/IAEC/2018/21).

## Author contributions

YR: Investigation, methodology, data curation, Writing – original draft. SS: Investigation, writing – review & editing. SP: Investigation, writing – review & editing. DS: Investigation. RS: Investigation. BR: Methodology. BD: Conceptualization, writing – review & editing. AB: Conceptualization, methodology, data curation, writing – review & editing, funding, and resource acquisition. All authors contributed to the article and approved the submitted version.
